# Learning to Swim

**DOI:** 10.4269/ajtmh.19-0473

**Published:** 2020-01

**Authors:** Zachary Tabb

**Affiliations:** Department of Pediatrics, Baylor College of Medicine, The Dr. Kelly DeScioli Global Child Health Residency Program, Houston, Texas

A person learns to swim in the water, not in a library. -Paulo Freire

With the doctor leading the way, arms clasped behind his back, the team entered. “I’m a former military man,” he reminded me, scanning the beds as though assessing his battalion. He wore a long white coat with the name Dr. Brightly over the right breast, a hand-me-down gift from a former visiting physician. I would later think of it as an amusingly ironic moniker for a man who generally carried himself around the hospital with a serious, nearly grave, countenance. Describing how he planned to mentor me, he warned, “outside the hospital I am your friend, but inside *there*,” pointing to the medical complex beside us, “I am your enemy.” Yet, in truth, his dour demeanor belied his passion for teaching, a welcoming nature, and a fondness for levity.

The head nurse entered next, followed by a handful of nursing students. Shifting aside, I was careful not to block their path as they hurried to deliver the paper charts from a bright blue-painted handcart. The blue color matched their scrubs and the same sky blue above holding that searing sun. The head nurse removed the first chart, handing it to the doctor.

As the attending physician read, the nurse presented the patient. “This patient arrived last night semi-conscious and at risk of convulsion. Her blood pressure was 100/60. Her temperature was 37.4°C. Lab results showed hemoglobin of 4.7. She was given a transfusion. Last week she gave birth and was discharged without complaints.” Sweat glistening on her forehead, the new mother strained to rise from bed. She looked young and was wrapped in an ornate and brilliantly colorful cloth (all the women were, in fact), made even brighter in contrast to the muted canvas of the walls surrounding us.

I thought about how before departing I received a basic pretravel orientation, supplementing this primer with additional reading from recommended tropical medicine texts. I understood my time would be limited and I wanted to hit the ground running. I felt excitement, and, undeservedly, a little confident.

It is one thing to read about healthcare disparities and entirely another to accompany, even briefly, the lives of those whose lived realities are defined by it. Statistics cannot convey the sight of patients lined in rows, shifting in creaking metal-framed beds. They do not transmit the disarming silence of a mother laboring inaudibly without anesthetic because of supply stockouts or the smell of sun-drying hospital linens and petrichor drifting through open-air hallways. One has to experience what is concealed by the numbers before learning can begin.

I realized the attending physician had asked me a question. “Do you know PPH? What does it mean? What are the causes? I know you are just a student and still early in your training, so you might not have heard this before. But I want you to *think*!” His eyes, normally ptotic and sleepy, bulged reflexively whenever he emphasized a point. “I want this hospital to pass through you, not you to pass through this hospital. You *get* me?” He had an endless armament of these turns of phrase, which he often packaged with rhetorical questions.

He began interviewing the patient in Twi, the local language, allowing me time to respond. I had moved to Apam, Ghana, between the first and second years of medical school to learn about the local management of HIV—the testing and treatment, community knowledge, and stigma. Intermixed with time spent conducting interviews with hospital staff and patients, I requested to participate on rounds and observe procedures in the operating room. I was intent on learning how care is delivered in a low-resource setting, a context where I one day hoped to live and work.

The maternity ward at this community hospital was sweltering and under the sudden attention in all new surroundings, I struggled to recall even the most basic medical facts. What I could recall seemed disconnected and irrelevant. Finally, I offered a cause of postpartum hemorrhage. With a tone that conveyed his doubts in my answer, he stopped me to ask, “Uh-huh, and what should you do for that?” After I proposed a solution, he would interject, “Uh-huh? the patient is still bleeding. *What else*?” It was clear he had no plans to relent.

Exhausted of ideas, I proposed sheepishly, “Apply gauze?” an answer that seemed so ridiculous as I said it that I nearly apologized. Before I could, he stopped writing and cast me a skeptical glance. “What would gauze do? No. I don’t agree with you.” And after more silent note-taking, without raising his eyes from the chart, he pressed further, “Uh-huh? I said I don’t agree with you.”

Now, 5 years later, as an intern in a global child health residency program, I will spend a year during my training at a hospital in a low-resource setting. Reflecting back to my time in Apam as an observer, “Dr. Brightly” pushed me to think through a clinical challenge, just as my attendings on the wards in the United States. As he expected me to learn the answer by the next day, I am held accountable similarly now. However, just as the settings are starkly different in their resources, so too can they be in their resourcefulness: in Apam I witnessed the critical role of the history and physical examinations in diagnosis, as well as teaching. We did not have the archives of the electronic medical record to reference further data. Nor did we have the imaging or laboratory technology that risks doing the thinking for us.

In contrast to the endless study-test cycles of my medical education, I never achieved the same grasp of the material as when I had to apply it to a patient in front of me. That is not to say you can skip reading (my evaluations, and Dr. Brightly, remind me of this fact). There is also value in the act of studying: the discipline, dedication, focus, and critical-thinking that develop over time. But I have only felt I *learned* once I began actually *doing*. The most instructive moments are those present with a patient exploring their story for clues, unlocking the secrets held by the physical examination, and considering questions about how access to and quality of healthcare tie it all together. These are the lessons I have retained over time, motivating me to press on, first as a student and now as a physician.

We continued along on rounds. In what felt like a moment of mercy, the attending recognized my limited knowledge about PPH, so he began to explain the common causes and interventions to address each. He instructed me to research it further as he would have additional questions tomorrow. Reaching the last room, in one of his frequent lessons, he paused to relate a clinical anecdote to the team. He remained standing, chin up, waiting for the moral to sink in. “Uh-huh? You know the import of the story?” he asked us, closing the last patient folder. “Never make your presence known, only your absence,” and without another word, he walked out of the room. We could hear him chuckling from down the hallway.

